# Reduced bone mineral density among HIV infected patients on anti-retroviral therapy in Blantyre, Malawi: Prevalence and associated factors

**DOI:** 10.1371/journal.pone.0227893

**Published:** 2020-01-14

**Authors:** Enock M. Chisati, Demitri Constantinou, Fanuel Lampiao

**Affiliations:** 1 Department of Physiotherapy, College of Medicine, University of Malawi, Blantyre, Malawi; 2 Consortium for Advanced Research Training in Africa (CARTA), Nairobi, Kenya; 3 Center for Exercise Science and Sports Medicine, FIMS Collaborating Center of Sports Medicine, University of the Witwatersrand, Johannesburg, South Africa; 4 Department of Biomedical Sciences, College of Medicine, University of Malawi, Blantyre, Malawi; National Institute for Communicable Disease (NICD), South Africa, SOUTH AFRICA

## Abstract

**Introduction:**

Use of tenofovir based anti-retroviral therapy (ART) in HIV patients is associated with low bone mineral density (BMD). Low BMD predisposes people living with HIV (PLWHIV) to fractures thereby increasing morbidity and mortality. Since the introduction of tenofovir based ARV regimens in 2011, information on the prevalence of low BMD in PLWHIV and receiving ART is still scarce in Malawi. This study aimed to determine the prevalence and associated factors of low BMD among adults living with HIV and receiving ART in Blantyre, Malawi.

**Methodology:**

This was a cross sectional study involving 282 HIV-positive adults of whom 102 (36%) were males. The participants aged 18–45 years were recruited from three primary and one tertiary health care facilities. Patients with no other comorbidities or conditions associated with low BMD and on ART >12 months were included. Data on BMD (femoral neck and lumbar spine) were collected using Dual–Energy X-ray Absorptiometry (DEXA). The International Physical Activity Questionnaire (IPAQ) was used to assess the physical activity (PA) levels. Participants’ body weight (kg) and height (m) were also measured. Descriptive statistics, Chi–Square test and multivariable logistic regression were used to analyse data.

**Results:**

Mean age of participants was 37(± 6.4) years, mean duration on ART was 5(± 3.5) years and mean body mass index (BMI) was 23(± 4.5) kg/m^2^. Twenty percent (55) had reduced BMD. More males (28%) had reduced BMD than females (14%) (*p* = 0.04). There was a significant association between lumbar BMD and femoral neck BMD (*r* = 0.66,*p*<0.001). However, on average, lumbar BMD (g/cm^2^) was significantly lower than the femoral BMD (*p* < 0.001). Participants with low PA level (*OR* 1.23,*p* = 0.6) had higher odds of having reduced BMD compared to those with high PA level.

**Conclusions and recommendation:**

Prevalence of reduced BMD is high among PLWHIV in Malawi especially male Malawian adults. Occurrence of low BMD is associated with low PA level. There is need for health care providers to routinely monitor BMD and PA levels of this population.

## Introduction

Bone mineral density (BMD) is a measure of bone strength as reflected by mineral content. Dual energy X-ray absorptiometry (DEXA) is globally accepted as a standard technique for measuring BMD performed typically at the lumbar spine and femoral neck[1]. BMD is assessed mostly to diagnose osteoporosis or osteopaenia which can predispose an individual to fractures thereby complicating morbidity and increasing the risk for mortality.[2].

Regardless of beneficial increases in survival, use of anti-retroviral therapy (ART) in people living with HIV (PLWHIV) is associated with low BMD[3–6]. An increased risk for hip fractures (hazard ratio, 6.2) among HIV infected patients compared to a non-HIV infected general population has been reported[7]. Compared to the risk of lung cancer (hazard ratio, 3.6) and a combined risk of cardiovascular and pulmonary diseases (odds ratio, 1.58), the risk for hip fractures is higher among people living with HIV [8,9]. Consequently, risk for mortality and morbidity in PLWHIV and receiving ART could increase due to the increasing risk for hip fractures.

Initiation of ART, irrespective of regimen, leads to increases in bone loss in PLWHIV [10,11]. A decrease of about 2–6% in BMD in the first two years after initiation of ART regardless of the regimen has been reported [12]. Although reductions in BMD occur at initiation of ART irrespective of regimen, tenofovir-based regimens are associated with more bone loss than other regimens [10,13–15]. Compared to other regimens, tenofovir leads to approximately 1–3% greater loss in BMD [10]. After comparing the effects of tenofovir and other ART regimens on BMD in PLWHIV, McComsey and colleagues observed greater decreases in BMD in patients receiving tenofovir-containing regimens than those receiving other regimens [15]. This could be suggestive of an independent effect of tenofovir on bone demineralisation regardless of host, viral and immunological factors. Although tenofovir has been shown to significantly contributes to reductions in bone mass [10,13–15], the World Health Organisation (WHO) recommends tenofovir-containing ART as first line treatment regimens in low income settings. [16,17]. This could therefore make reduced BMD highly likely among PLWHIV in low income settings [13].

Higher prevalence rates of up to 85% of low BMD among PLWHIV in low and middle income countries have been reported by a number of studies[18–22]. Apart from ART, factors such as lack of physical activity (PA), lower body mass index (BMI), female sex, older age, nutritional deficiencies of calcium and vitamin D, depression, contraception use, smoking and alcohol use are believed to contribute to high prevalence of low BMD among PLWHIV[22–25]. Although most of the risk factors are similar in low income and high income settings[26], some risk factors such as malnutrition, low BMI and longer duration without ART treatment after the diagnosis of HIV are more common among PLWHIV in low and middle income settings[13]. These factors could make high prevalence rates of reduced BMD unavoidable among PLWHIV in low and middle income countries.

Malawi’s HIV prevalence is one of the highest in the world and accounts for 4% of the total number of PLWHIV in sub-Sahara Africa[27]. About 9.2% of the adult population aged between 15 and 49 are living with HIV in Malawi[27]. In line with WHO recommendations [16], Malawi introduced tenofovir based ARTs in 2011 as first line treatment regimen for PLWHIV[28]. Despite this, there has been minimum effort to investigate the burden of low BMD in such patients in Malawi. In view of the foregoing, this study aimed to determine the prevalence and associated factors of low BMD among PLWHIV on ART in Blantyre, Malawi.

## Methods

### Study setting

This study was conducted at three primary health care facilities (Limbe Health Center, Gateway Health Center and Dream Center) and one tertiary health care facility (Queen Elizabeth Central Hospital, QECH) located within Blantyre city, Malawi. The health facilities are located in four different townships of Limbe, Mandala, Chinyonga and Ginnery Corner in Blantyre (one health facility per township). Limbe Health Center, Gateway Health Center and QECH are public health facilities that run daily ART clinics for PLWHIV from the surrounding areas. Dream Center on the other hand, is a private health care facility that runs ART clinics three days of the week. All four health care facilities provide ART medication refill for PLWHIV on an out-patient basis. Each day the three public health facilities provide ART refills to approximately 50 PLWHIV, whereas Dream Center serves approximately 25 patients. All four health care facilities have medical consultants supported by nurses who provide ART services to the patients.

### Study design and population

This was a cross sectional study involving 282 male and female adults aged 18 years to 45 years and receiving ART at the four health facilities. A random sample of four from a list of 10 health facilities in Blantyre city was selected using the RANDBETWEEN function in Microsoft Excel 2016. Using consecutive sampling, a proportional sample was obtained from each facility. Data were collected from February, 2018 to March, 2019.

**Fig 1:**
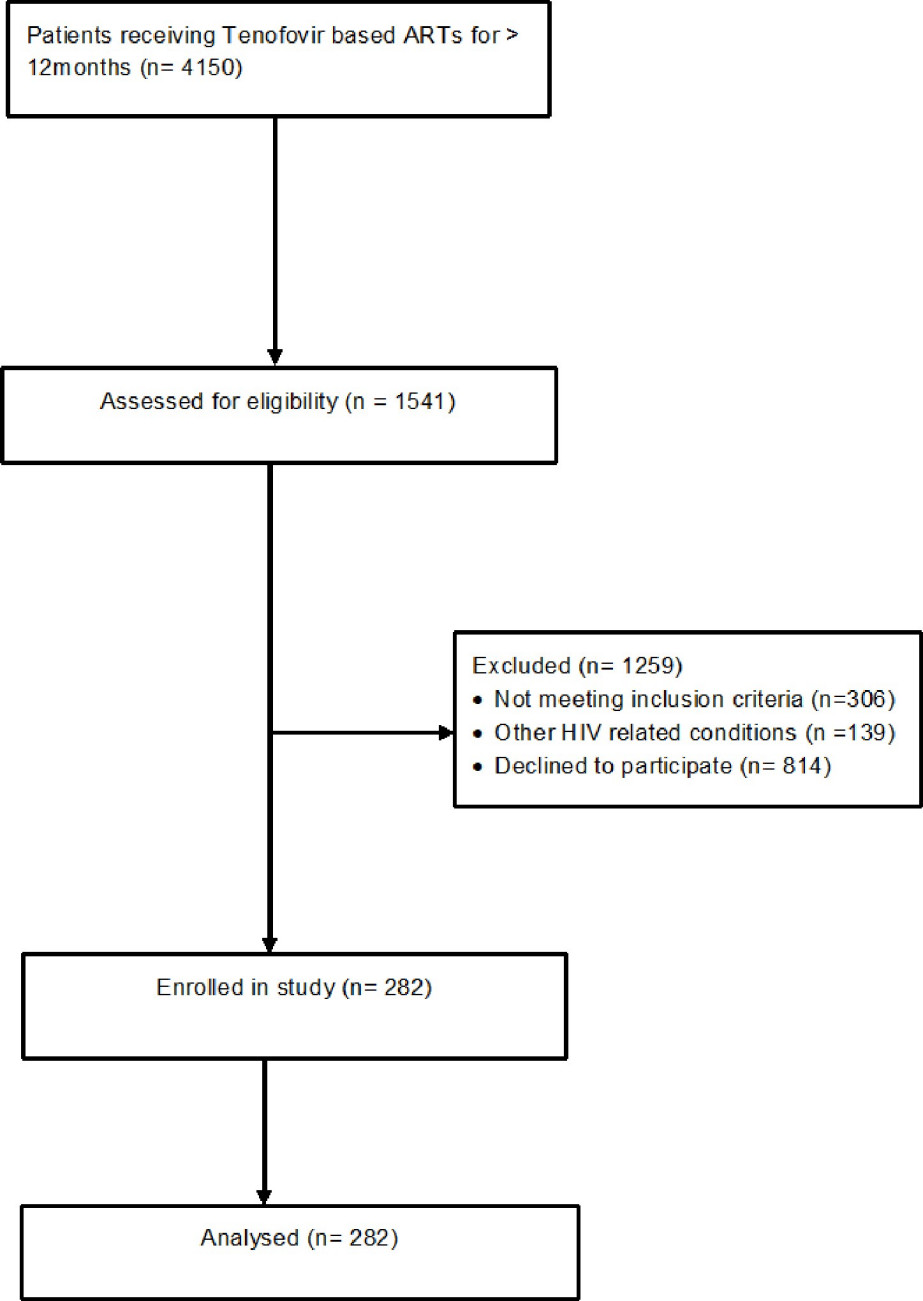
Study flow diagram.

Patients were invited to participate and after signing informed consent, were included if they were receiving tenofovir based ART regimens for at least 12 months. Participants who had been on ART for at least 12 months were selected as reductions in BMD are more pronounced after one year[12]. Those with a history of diabetes mellitus or impaired glucose tolerance, an active acute opportunistic infection, rheumatoid arthritis, severe diarrhoea, tuberculosis within one month of commencing treatment, glucocorticoid therapy within the past six months, currently pregnant, breast feeding women, contraception medication use or known to be in renal failure, were excluded because such conditions contribute to reduced BMD[22] (Fig 1).

### Data collection and tools

#### Demographic data

Trained health workers obtained socio-demographic data from the recruited participants. Data on participants’ information on smoking (yes/no), alcohol (yes/no), calcium diet measured as milk intake more than once per week (yes/no) and traditional medication use (yes/no) was obtained using a data collection form. Data on age (years), ART duration and regimen were obtained from participants’ clinical records.

#### Height and body weight

Body weight (kg) and height (cm) measurements were obtained using a Stadiometer (HS–DBS00361, Model: 1127154) following manufacturer guidelines. Body mass index (BMI) for each participant was calculated by dividing weight measurement by the square of the height measurement in meters (m^2^).

#### Physical activity

The International Physical Activity Questionnaire (IPAQ) was used to collect data on PA levels of the participants. The IPAQ comprised questions on frequency, intensity and duration of PA that participants do as part of their everyday life in the previous 7 days. Questions such as “During the last 7 days, on how many days did you do vigorous physical activities like heavy lifting, digging, gardening, aerobics, or fast bicycling?”; “How much time did you usually spend doing vigorous physical activities on one of those days?”; “During the last 7 days, on how many days did you do moderate physical activities like carrying light loads, bicycling at a regular pace, or participating in light sporting events?”; “How much time did you usually spend doing moderate physical activities on one of those days?” etc were asked. To ensure that the questions in the local language (Chichewa) were conceptually equivalent with the English questions in the IPAQ, forward and backward translation was performed by independent translators. The IPAQ was proven to be a valid and reliable tool for measuring PA among adults aged 18–65 years in diverse settings[29].

Variable minutes spent on doing PA were recorded. The minutes were calculated into metabolic equivalents (METs). METs are defined as multiples of the resting metabolic rate (1 *MET* = 3.5*ml O*_2_ *kg*^−1^ *min*^−1^) and MET-minutes were calculated by multiplying the MET score of an activity (an equivalence of kilocalories for a 60 kg person) by the minutes performed[30].

Participants were categorized into low, moderate and high PA levels. Low level PA comprised participants having the lowest PA and did not meet the criteria for moderate or high PA levels. Moderate PA level comprised participants who did 3 or more days of vigorous PA for at least 20 minutes per day. Similarly, participants who performed 5 or more days of walking or moderate intensity PA for at least 30 minutes per day, fell into the moderate PA level category. High PA level comprised participants who performed at least 3 days of vigorous intensity PA accumulating at least 1500 MET minutes per week. Similarly, participants who performed 7 or more days of any combination of vigorous PA, moderate intensity PA or walking achieving a total of at least 3000 MET minutes per week, fell into the high PA level category.

#### Bone mineral density

All BMD measurements were performed at QECH. Femoral neck and lumbar spine bone mineral density was measured using DEXA [Hologic Discovery-Wi (S/N 84668), software version 13.5.3.2:5, Hologic Bedford Inc., Bedford, MA, USA]. Femoral neck BMD was measured at the left hip. Measurements of unilateral femoral neck BMD minimizes radiation exposure associated with radiography, time, as well as medical costs[31]. Lumbar spine BMD was measured from the first to the fourth lumbar spines.

Z-scores were obtained as an outcome measure of BMD. The World Health Organisation (WHO) recommends the use of Z-scores (defined as an individuals’ BMD in comparison to age-matched normal individuals) in reporting BMD for premenopausal women or men less than 50 years of age, and children[2]. A Z-score of −2.0 or lower is defined as low BMD for chronological age or below the expected range for age whereas a Z-score above −2.0 is within the expected range for age[32–34]. Participants were categorised as having low BMD if the femoral neck or total lumbar spine Z-score was– 2 or less.

### Data analysis

Data were analysed using IBM Statistical Package for the Social Sciences (SPSS) version 21. Descriptive statistics using mean and standard deviation (SD) were used to characterize age, height, weight and BMI variables. Data for continuous variables were normally distributed. Student T–tests were used to analyse the differences between lumbar spine and femoral neck BMD. Chi–Square was used to test associations between categorical variables and BMD. Simple linear regression was used to analyse correlations between lumbar BMD and femoral neck BMD. A logistic regression model was fitted to assess effects of PA, smoking, alcohol, calcium diet and traditional medicine on low BMD. Variables for inclusion into the multivariable models were selected based on biological plausibility and those variables with p≤0.20 at bivariable level were taken for multivariable analysis. Odds ratios (OR) were obtained to quantify the probability of having low BMD. All statistical tests were two—sided and p values of ≤ 0.05 were considered statistically significant.

### Ethical considerations

As participants came for refilling their ART medication, they were requested to attend a health talk regarding the purpose of the study and requesting their participation. This health talk was conducted by the researchers and the health workers at the clinic. After having their ART refill, willing participants were directed into a separate room where the aim and objectives of the study were again explained and screening for eligibility was done. Consent was obtained from eligible and willing participants. All ethical procedures were followed and privacy and confidentiality were ensured by allocating codes to the participants. The study was approved by the University of Malawi’s College of Medicine Research and Ethics Committee (COMREC) registration number P.06/17/2206.

## Results

A total of 282 adult people living with HIV and receiving tenofovir based ART were included in the study. Height was significantly higher in males than females whereas BMI was significantly higher in females than males (P = < 0.001, two sample T–test) (Table 1). Out of 282 participants, 55 (20%) had low BMD while 227 (80%) had their BMD within the expected ranges for age. More males (28%) had reduced BMD than females (14%) with most of the reduced BMD observed in the lumbar spine. The proportion of participants with reduced BMD was significantly higher among males compared to females (28% vs 14%, P = 0.04, Chi–Square test) (Table 1).

**Table 1:** Characteristics of participants.

	Total n = 282	Male n = 102	Female n = 180	P Value
Age (yrs)	37 ± 6.4	37 ± 6.5	37 ± 6.2	0.49
Weight (kg)	60 ± 11.2	61 ± 10.2	60 ± 12.1	0.84
Height (cm)	160 ± 0.1	165 ± 0.1	158 ± 0.1	< 0.001*
BMI (kg/m^2^)	23 ± 4.5	22 ± 3.3	24 ± 4.9	< 0.001*
ART duration (yrs)	5.3±3.5	5.2±3.6	5.4±3.4	0.72
BMD within expected range for age [*n*(%)]	227 (80)	73 (72)	154 (86)	
BMD below expected range for age [*n*(%)]	55 (20)	29 (28)	26 (14)	0.04≠

Data are in mean ± SD; BMI = body mass index

* = statistically significant

^≠^ = Chi-square test

**Fig 2:**
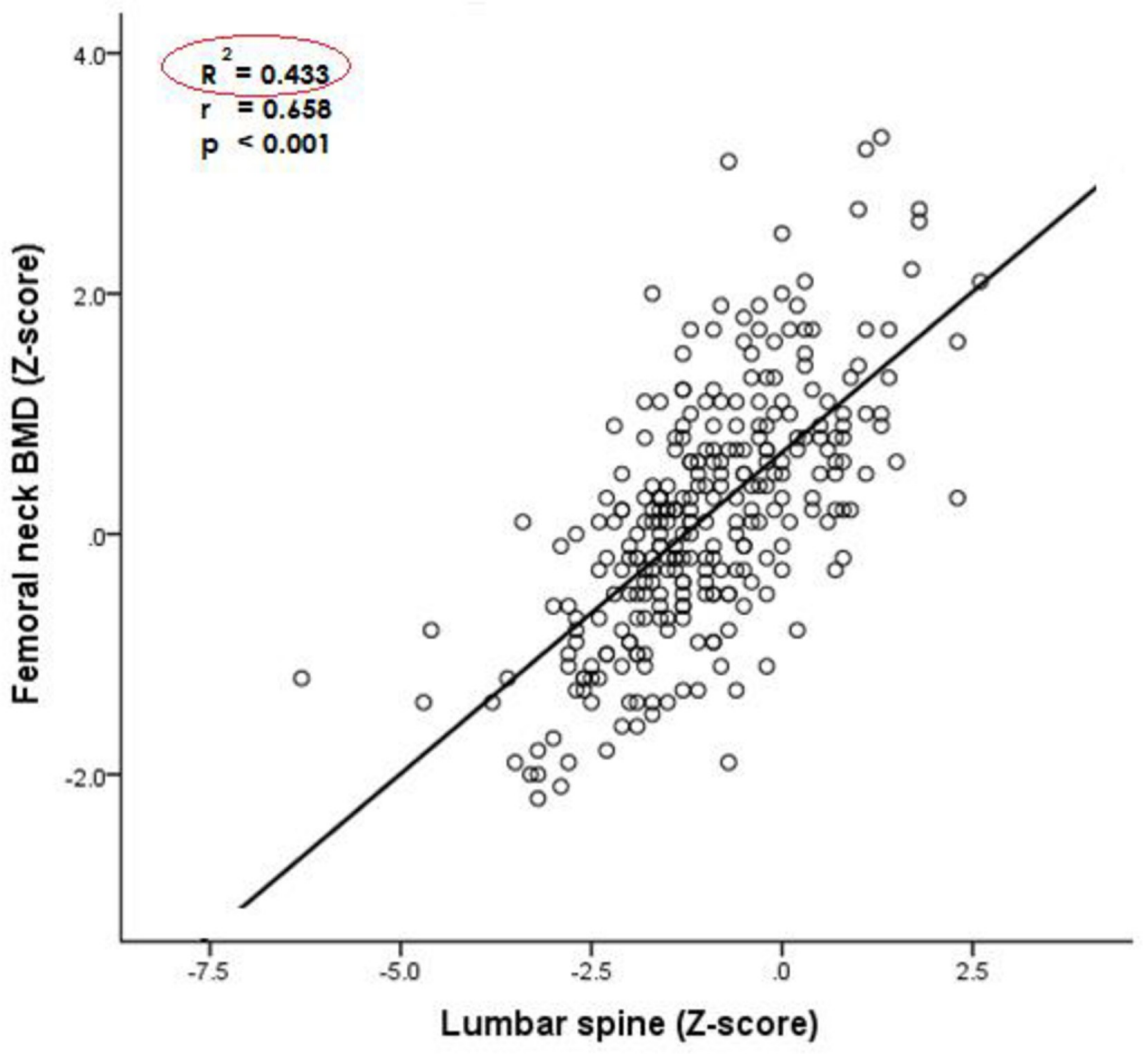
Association between femoral neck and lumbar bone mineral density.

There was a significant positive association between lumbar BMD and femoral neck BMD (*r* = 0.66, *P*<0.001). Fourty three percent of participants had reduced BMD in both the lumbar spine and femoral neck (R^2^ = 0.43) (Fig 2).

On average, lumbar BMD (g/cm^2^) was significantly lower compared to femoral BMD among all participants (mean difference– 0.08, *P* < 0.001, Paired T–test) (Table 2).

**Table 2:** Differences between femoral neck and lumbar bone mineral density.

Bone parameter	Lumbar spine	Femoral neck	Mean difference	95% CI	P–Value
BMD (g/cm^2^)	0.93	0.86	– 0.08	– 0. 09 to– 0.07	< 0.001*
Z–Score	– 1.01	0.14	1.15	1.03 to 1.26	< 0.001*

* = statistically significant

Participants with low PA level (OR 1.23, p = 0.6) were more likely to have reduced BMD than those with high PA level. Participants who were not consuming a calcium diet were less likely to have reduced BMD despite a significant association between no calcium diet and low BMD (OR 0.38, p = 0.004) (Table 3). Smoking, alcohol and traditional medicine use were not significantly associated with the occurrence of low BMD.

**Table 3:** Factors associated with low BMD.

Factor		Adjusted OR	95% CI	P value
PA level				
	High	1(ref)		
	Moderate	0.87	0.41–1.85	0.72
	Low	1.23	0.57–2.67	0.6
Calcium				
	Yes	1(ref)		
	No	0.38	0.19–0.74	0.004
Smoking				
	No	1(ref)		
	Yes	0.57	0.13–2.56	0.47
Alcohol				
	No	1(ref)		
	Yes	0.88	0.33–2.34	0.8
Traditional Medicine				
	No	1(ref)		
	Yes	0.38	0.11–1.36	0.14

(ref) = reference variable

## Discussion

The main purpose of the study was to investigate the prevalence of reduced BMD and associated factors among Malawian adults living with HIV and receiving tenofovir based ART. Findings of the study reveal a high prevalence of reduced BMD among PLWHIV and receiving ART in Malawi. Findings also show that the occurrence of reduced BMD is associated with low PA level.

A comparable prevalence in reduced BMD (23%) was found by Cardeal *et al* (2017) among 108 individuals living with HIV in Brazil[23]. However, some studies have reported higher prevalence rates of reduced BMD ranging from 37% to 80% among PLWHIV [18–20,35]. A slightly lower prevalence in reduced BMD revealed in this study could be due to the age group of participants. The current study recruited adults living with HIV between 18 years and 45 years with a mean age of 37 years. Studies that reported higher prevalence in reduced BMD included participants older than 55 years whose bone mass may have already started decreasing due to ageing. Bone mass increases with age and peaks between ages 25 and 30 years, thereafter bone mass starts to decrease leading to low BMD[36]. After 30 years of age, BMD is maintained for about 10 years before it starts to decline at a rate of about 0.3–0.5% per year in both males and females[37,38]. At ages between 45 to 55 women lose more bone mineral than men after which the rate of bone loss is gradual and the same in both sexes[38]. A rapid loss of BMD in women between ages 45 to 55 is attributed to decreases in oestrogen production as the menstrual cycle ceases during this period[37]. Therefore, a slightly lower prevalence in reduced BMD among PLWHIV revealed in the current study compared to other studies could be attributed to bone strength associated with younger age of most of the participants.

Notably, more males in the current study had reduced BMD than females with most of the reduced bone mass observed in the lumbar spine. Despite comparable body weight, evidence on bone physiology indicate that males have more bone mass than females due to differences in skeletal muscle mass and the body’s response to changes in hormones[39,40]. In line with bone physiology, a recent study by Erlandson *et al* (2018) has reported BMD reduction twice as quickly among HIV infected women compared with men[41]. However, 24% of female participants in the study by Erlandson *et al* were menopausal thus leading to more BMD decline[41]. Pronounced decreases in BMD among women has been attributed to large decreases in oestrogen during menopause[39]. Contrary to findings by Erlandson *et al*, the current study did not include female participants in menopause. Pronounced decreases in BMD at lumbar spine among males found in the current study could be due to lifestyle activities among Malawians. In Malawi most females, as opposed to males, carry loads on their heads which likely impacts the lumbar spine which could in turn lead to high density in lumbar spine bones.

Consistent with other studies[42,43], a significant positive correlation between lumbar spine and femoral neck BMD was revealed in the current study. Closer to half of participants who had reduced BMD in the femoral neck were also likely to have reduced BMD in the lumbar spine. With the significant level of correlation, findings of the current study suggest that lumbar spine BMD could be used to predict femoral neck BMD among PLWHIV and receiving ART in Malawi.

Contrary to the current findings, some studies have reported greater reductions in femoral BMD than lumbar BMD among people living with HIV[35,41,44]. However, these studies reporting more reduced BMD in the femoral neck than the lumbar spine were conducted among western and Asian populations who have different PA lifestyles compared to Sub-Saharan African populations. Since bone strength depends, in part, on mechanical stress or mechanical loading, an individual’s physical activity lifestyle can play a role in either increasing or reducing BMD[37]. Lower BMD in the lumbar spine compared to the femoral neck observed in the current study could be due to PA of Malawians such as walking long distances to work[45] which may impact the femoral neck bone thereby increasing BMD.

The likely occurence of reduced BMD among participants with low PA level revealed in this study is supported by growing evidence that demonstrates a negative impact of low PA on BMD[24]. Guidelines for good bone health recommends PA as an important component in preventing bone loss among PLWHIV[46]. As demonstrated by Santo et al (2015), significant improvements in BMD were revealed among 20 PLWHIV after particpating in a 12 weeks strength exercise programme[47]. Suggesting that PA could play a role in mainting BMD among PLWHIV. Current findings supports foundation evidence for considering PA as an intervention for managing reduced BMD in PLWHIV.

Being the first in Malawi, this study adds to the body of knowledge on prevalence of reduced BMD among PLWHIV in resource limited settings. The study also provides valuable information on PA and bone health for Malawian adults living with HIV and receiving ART. On the other hand, the current study used a subjective approach of obtaining PA levels through self-reported questionnaire which may result in reporting bias. In addition, the change of BMD over the period of treament could not be established due to the cross sectional design of this study. In addition, most references in the current study reflect circumstances in western countries due to lack of BMD data on large age related healthy African cohorts that could be used for refernce. Furthermore, the current study did not collect data on viral loads or CD4 cell count to determine ART adherence and the cumulative effect of tenofovir exposure among those with reduced BMD. Longitudinal studies aimed at evaluating BMD dynamics in relation to PA are therefore warranted.

## Conclusions and recommendations

Reduced BMD is prevalent among Malawian adults living with HIV and receiving ART. Among those with reduced BMD, males had more bone loss than females which may contribute to more incidences of stress and osteoporotic fractures among adult Malawian males living with HIV. Most of the BMD among males was observed at the lumbar spine. The occurrence of reduced BMD was more likely in those who had low PA level. There is need for health care providers to routinely monitor BMD and PA level of this population. In addition, there is need for further research on the effects of exercise interventions on BMD among PLWHIV.

## References

[1] Dimai HP. Use of dual-energy X-ray absorptiometry (DXA) for diagnosis and fracture risk assessment; WHO-criteria, T- and Z-score, and reference databases. Bone [Internet]. 2016;1–5. Available from: 10.1016/j.bone.2016.12.016 28041872

[2] World Health Organisation. Scientific group on the assessment of osteoporosis at primary health care level. In: Summary Meeting Report. 2004. p. 1–13.

[3] Mirembe BG, Kelly CW, Mgodi N, Greenspan S, Dai JY, Mayo A, et al Bone Mineral Density Changes Among Young, Healthy African Women Receiving Oral Tenofovir for HIV Preexposure Prophylaxis. J Acquir Immune Defic Syndr [Internet]. 2016;71(3):287–94. Available from: http://content.wkhealth.com/linkback/openurl?sid=WKPTLP:landingpage&an=00126334-201603010-00007 10.1097/QAI.0000000000000858 26866954 PMC4755358

[4] Dave JA, Cohen K, Micklesfield LK, Maartens G, Levitt NS. Antiretroviral Therapy, Especially Efavirenz, Is Associated with Low Bone Mineral Density in HIV-Infected South Africans. PLoS One [Internet]. 2015;10(12):1–9. Available from: 10.1371/journal.pone.0144286 PMC4669137 26633015

[5] Liu AY, Vittinghoff E, Sellmeyer DE, Irvin R, Mulligan K, Mayer K, et al Bone mineral density in HIV-negative men participating in a tenofovir pre-exposure prophylaxis randomized clinical trial in San Francisco. PLoS One. 2011;6(8). 10.1371/journal.pone.0023688 PMC3163584 21897852

[6] Purday J, Gafni R, Reynolds J, Zeichner S, Hazra R. Decreased bone mineral density with off—label use of Tenofovir in HIV—Infected children and adolescents. J Paediatr. 2008;152(4):582–4. 10.1016/j.jpeds.2007.12.020 PMC2390888 18346519

[7] Güerri‐Fernandez R, Vestergaard P, Carbonell C, Knobel H, Avilés FF, Castro AS, et al HIV Infection Is Strongly Associated With Hip Fracture Risk, Independently of Age, Gender, and Comorbidities: A Population‐Based Cohort Study. J Bone Miner Res. 2013;28(6):1259–63. 10.1002/jbmr.1874 23362011

[8] Kirk GD, Merlo C, Driscoll PO, Mehta SH, Galai N, Vlahov D, et al HIV Infection Is Associated with an Increased Risk for Lung Cancer, Independent of Smoking. Clin Infect Dis. 2007;45(1):103–10. 10.1086/518606 17554710 PMC4078722

[9] Schouten J, Wit FW, Stolte IG, Kootstra NA, Van Der Valk M, Geerlings SE, et al Cross-sectional comparison of the prevalence of age-associated comorbidities and their risk factors between hiv-infected and uninfected individuals: The age H IV cohort study. Clin Infect Dis. 2014;59(12):1787–97. 10.1093/cid/ciu701 25182245

[10] Grant PM, Cotter AG. Tenofovir and bone health. Curr Opin HIV AIDS. 2016;11(3):326–32. 10.1097/COH.0000000000000248 26859637 PMC4844450

[11] Yin MT, Overton ET. Increasing clarity on bone loss associated with antiretroviral initiation. J Infect Dis. 2011;203(12):1705–7. 10.1093/infdis/jir184 21606527

[12] Duvivier C, Kolta S, Assoumou L, Ghosn J, Rozenberg S, Murphy RL, et al Greater decrease in bone mineral density with protease inhibitor regimens compared with nonnucleoside reverse transcriptase inhibitor regimens in HIV-1 infected naive patients. AIDS. 2009;23(7):817–24. 10.1097/QAD.0b013e328328f789 19363330

[13] Matovu FK, Wattanachanya L, Beksinsk M, Pettifor JM, Ruxrungtham K. Bone health and HIV in resource-limited settings: a scoping review. Curr Opin HIV AIDS [Internet]. 2016;11(3):306–25. Available from: http://www.ncbi.nlm.nih.gov/pubmed/27023284 10.1097/COH.0000000000000274 27023284 PMC5578733

[14] Brown TT, Moser C, Currier JS, Ribaudo HJ, Rothenberg J, Kelesidis T, et al Changes in bone mineral density after initiation of antiretroviral treatment with Tenofovir Disoproxil Fumarate/Emtricitabine Plus Atazanavir/Ritonavir, Darunavir/Ritonavir, or Raltegravir. J Infect Dis. 2015;212(8):1241–9. 10.1093/infdis/jiv194 25948863 PMC4577040

[15] McComsey GA, Kitch D, Daar ES, Tierney C, Jahed NC, Tebas P, et al Bone mineral density and fractures in antiretroviral-naive persons randomized to receive abacavir-lamivudine or tenofovir disoproxil fumarate-emtricitabine along with efavirenz or atazanavir-ritonavir: AIDS Clinical Trials Group A5224s, a substudy of ACTG. J Infect Dis. 2011;203(12):1791–801. 10.1093/infdis/jir188 21606537 PMC3100514

[16] World Health Organisation. Consolidated guidelines on the use of antiretroviral drugs for treating and preventing HIV infection. In: Guidelines. 2013 p. 1–269. 24716260

[17] World Health Organisation. Consolidated Guidelines on the Use of Antiretroviral Drugs for Treating and Preventing HIV Infection: What ‘ S New. In: Policy Brief. 2015 p. 1–16.

[18] Alonge T, Okoje-Adesomoju V, Atalabi O, Obamuyide H, Olaleye D, Adewole I. Prevalence of abnormal bone mineral density in HIV-positive patients in ibadan, Nigeria. J West African Coll Surg. 2013;3(4):1–14. PMC4437238 26046022

[19] Chitu-Tisu CE, Barbu EC, Lazar M, Ion DA, Badarau IA. Low bone mineral density and associated risk factors in HIV-infected patients. GERMS [Internet]. 2016;6(2):50–9. Available from: http://www.ncbi.nlm.nih.gov/pubmed/27482514 10.11599/germs.2016.1089 27482514 PMC4956161

[20] Dravid A;, Kulkarni M, Borkar A, Dhande Sachin. Prevalence of low bone mineral density among HIV patients on longterm suppressive antiretroviral therapy in resource limited setting of western India. J Int AIDS Soc. 2014;17(November):17–8. 10.7448/IAS.17.4.19567 PMC4224871 25394074

[21] Aydin OA, Karaosmanoglu HK, Karahasanoglu R, Tahmaz M, Nazlican O. Prevalence and risk factors of osteopenia/osteoporosis in Turkish HIV/AIDS patients. Brazilian J Infect Dis [Internet]. 2013;17(6):707–11. Available from: 10.1016/j.bjid.2013.05.009 PMC9427423 24076108

[22] Pinto Neto LFS, Ragi-Eis S, Vieira NFR, Soprani M, Neves MB, Ribeiro-Rodrigues R, et al Low Bone Mass Prevalence, Therapy Type, and Clinical Risk Factors in an HIV-Infected Brazilian Population. J Clin Densitom. 2011;14(4):434–9. 10.1016/j.jocd.2011.06.004 22051092

[23] Cardeal D, Soares LR, Pereira RMR, Rutherford GW, Assone T, Takayama L, et al Low bone mineral density among HIV infected patients in Brazil. J Sao Paulo Inst Trop Med. 2017;59(e89):1–5. 10.1590/S1678-9946201759089 PMC5738774 29267597

[24] Perazzo JD, Webel AR, Alam SMK, Sattar A, McComsey GA. Relationships Between Physical Activity and Bone Density in People Living with HIV: Results from the SATURN-HIV Study. J Assoc Nurses AIDS Care. 2018;29(4):528–37. 10.1016/j.jana.2018.03.004 29735237 PMC5999576

[25] Welz T, Childs K, Ibrahim F, Poulton M, Taylor CB, Moniz CF, et al Efavirenz is associated with severe vitamin D deficiency and increased alkaline phosphatase. AIDS [Internet]. 2010;24(12):1923–8. Available from: http://www.ncbi.nlm.nih.gov/entrez/query.fcgi?cmd=Retrieve&db=PubMed&dopt=Citation&list_uids=20588161 10.1097/QAD.0b013e32833c3281 20588161

[26] Bonjoch A, Figueras M, Estany C, Perez-Alvarez N, Rosales J, del Rio L, et al High prevalence of and progression to low bone mineral density in HIV-infected patients: a longitudinal cohort study. AIDS [Internet]. 2010;24(18):2827–33. Available from: http://content.wkhealth.com/linkback/openurl?sid=WKPTLP:landingpage&an=00002030-201011270-00009 10.1097/QAD.0b013e328340a28d 21045635

[27] UNIAIDS. Global AIDS Monitoring 2018. 2017.

[28] Malawi Government. Global AIDS Response Progress Report (GARPR): Malawi Progress Report for 2013. 2014.

[29] Bassett DR. International physical activity questionnaire: 12-Country reliability and validity. Med Sci Sports Exerc. 2003;35(8):1396 10.1249/01.MSS.0000078923.96621.1D 12900695

[30] IPAQ Research Committee. Guidelines for Data Processing and Analysis of the International Physical Activity Questionnaire (IPAQ)–Short and Long Forms. Ipaq. 2005.

[31] Lessig HJ, Meltzer MS, Siegel JA. The symmetry of hip bone mineral density. A dual photon absorptiometry approach. Clin Nucl Med. 1987;12(10):811–2. 10.1097/00003072-198710000-00013 3677526

[32] International Society for Clinical Densitometry. 2013 Official Positions—Adult. http://www.iscd.org/official-positions/2013-iscd-official-positions-adult/. 2013.

[33] Cosman F, de Beur SJ, LeBoff MS, Lewiecki EM, Tanner B, Randall S, et al Clinician ‘ s Guide to Prevention and Treatment of Osteoporosis. Osteoporos Int. 2014;25:2359–81. 10.1007/s00198-014-2794-2 25182228 PMC4176573

[34] Kanis JA. Diagnosis of osteoporosis and assessment of fracture risk. Lancet. 2002;359(June 1):1929–36. 12057569 10.1016/S0140-6736(02)08761-5

[35] Escota G V., Mondy K, Bush T, Conley L, Brooks JT, Önen N, et al High Prevalence of Low Bone Mineral Density and Substantial Bone Loss over 4 Years Among HIV-Infected Persons in the Era of Modern Antiretroviral Therapy. AIDS Res Hum Retroviruses [Internet]. 2015;31(1):59–67. Available from: http://online.liebertpub.com/doi/10.1089/aid.2015.0158%5Cnhttp://www.ncbi.nlm.nih.gov/pubmed/26366785 10.1089/aid.2015.0158 26366785

[36] Seeman E, Delmas PD. Bone quality—the material and structural basis of bone strength and fragility. N Engl J Med. 2006;354(21):2250–61. 10.1056/NEJMra053077 16723616

[37] Kruger MJ, Nell TA. Bone mineral density in people living with HIV: a narrative review of the literature. AIDS Res Ther [Internet]. 2017;14(35):1–17. Available from: http://aidsrestherapy.biomedcentral.com/articles/10.1186/s12981-017-0162-y 28747190 10.1186/s12981-017-0162-y PMC5530558

[38] Baxter-Jones ADG, Faulkner RA, Forwood MR, Mirwald RL, Bailey DA. Bone mineral accrual from 8 to 30 years of age: An estimation of peak bone mass. J Bone Miner Res. 2011;26(8):1729–39. 10.1002/jbmr.412 21520276

[39] Lang TF. The Bone-Muscle Relationship in Men and Women. J Osteoporos. 2011;2011:1–4. 10.4061/2011/702735 PMC3189615 22007336

[40] Nieves JW, Formica C, Ruffing J, Zion M, Garrett P, Lindsay R, et al Males Have Larger Skeletal Size and Bone Mass Than Females, Despite Comparable Body Size. J Bone Miner Res. 2005;20(3):529–35. 10.1359/JBMR.041005 15746999

[41] Erlandson KM, Lake JE, Sim M, Falutz J, Prado CM, Domingues R, et al Bone Mineral Density Declines Twice as Quickly Among HIV-Infected Women Compared With Men. J Acquir Immune Defic Syndr. 2018;77(3):288–94. 10.1097/QAI.0000000000001591 29140875 PMC5807215

[42] Choe HS, Lee JH, Min DK, Shin SH. Comparison of vertebral and femoral bone mineral density in adult females. J Phys Ther Sci. 2016;28:1928–31. 10.1589/jpts.28.1928 27390449 PMC4932090

[43] Namwongprom S, Ekmahachai M. Bone Mineral Density: Correlation between the Lumbar Spine, Proximal Femur and Radius in Northern Thai Women. J Med Assoc Thail. 2011;94(6):725–31. 21696083

[44] Zhang L, Su Y, Hsieh E, Xia W, Xie J, Han Y, et al Bone turnover and bone mineral density in HIV-1 infected Chinese taking highly active antiretroviral therapy -a prospective observational study. BMC Musculoskelet Disord. 2013;14:224 10.1186/1471-2474-14-224 23899016 PMC3734166

[45] Guthold R, Louazani SA, Riley LM, Cowan MJ, Bovet P, Damasceno A, et al Physical Activity in 22 African Countries: Results from the World Health Organization STEPwise Approach to Chronic Disease Risk Factor Surveillance. Am J Prev Med. 2011;41(1):52–60. 10.1016/j.amepre.2011.03.008 21665063

[46] Borderi M, Pierluigi V. How to monitor bone disease in HIV infection. HAART, HIV Correl Pathol other Infect. 2013;182–9.

[47] Santos W, Santos W, Paes P, Ferreira-Silva I, Santos A, Vercese N, et al Impact of Strength Training on Bone Mineral Density in Patients Infected with HIV exhibiting Lipodystrophy. J Strength Cond Res. 2015;29(12):3466–71. 10.1519/JSC.0000000000001001 25970490

